# Quality of Life in Burn Survivors Post-Discharge: A Narrative Review

**DOI:** 10.3390/medicina62071218

**Published:** 2026-06-23

**Authors:** Andreea Ungureanu, Adriana-Nicoleta Trandafir, Maria-Cristina Marinescu, Valeria Coviltir, Carmen Giuglea, Silviu-Adrian Marinescu

**Affiliations:** 1 Doctoral School, Carol Davila University of Medicine and Pharmacy, 8 Eroii Sanitari Bvd., 050474 Bucharest, Romania; andreea.ungureanu2020@drd.umfcd.ro; 2 Department of Plastic and Reconstructive Surgery, ‘Bagdasar-Arseni’ Emergency Hospital, 12 Berceni Rd., 041915 Bucharest, Romania; 3 Department of Plastic and Reconstructive Surgery, ‘Carol Davila’ Central Military Emergency Hospital, 88 Mircea Vulcănescu St., 10825 Bucharest, Romania; 4 Discipline of Medical Physiology, Faculty of Medicine, Carol Davila University of Medicine and Pharmacy, 020021 Bucharest, Romania; 5 Department of Ophthalmology, Faculty of Medicine, Carol Davila University of Medicine and Pharmacy, 050474 Bucharest, Romania; 6 Department of Plastic and Reconstructive Surgery, Carol Davila University of Medicine and Pharmacy, 8 Eroii Sanitari Bvd., 050474 Bucharest, Romania

**Keywords:** post-burn quality of life, social reintegration, return to work, long-term outcomes, quality of life trajectory

## Abstract

Burn injuries are increasingly recognized as chronic conditions with enduring physical, psychological and social consequences that extend beyond acute survival. This narrative review synthesizes and interpretatively discusses recent evidence on health-related quality of life (HRQoL) in adult burn survivors, focusing on recovery patterns following discharge. Persistent physical sequelae—particularly chronic pain, pruritus, contractures and scarring—remain major determinants of reduced HRQoL, mainly mediated by functional limitation and self-perception. Psychological morbidity is common, with high rates of depression, anxiety and post-traumatic stress disorder, particularly early after injury, although post-traumatic growth may also emerge. Social reintegration, including return to work, is often delayed or incomplete and is influenced by injury severity, mental health status and social support. Recovery trajectories are nonlinear: the greatest improvements occur within the first six months, followed by slower gains up to 18–24 months, after which many patients fail to reach population norms. Pain and psychological symptoms may persist for years. Overall, these findings support a multidisciplinary, longitudinal approach to burn care, emphasizing early risk stratification and rehabilitation to optimize individualized recovery. In this narrative review, we aim to outline the main dimensions of long-term quality of life, with a particular focus on the temporal dynamics of recovery patterns.

## 1. Introduction

In recent years, the aim of burn care has shifted from a focus on survival toward addressing the consequences of injury, emphasizing that the quality of the outcome must be worth the pain of survival [1]. Traditionally, research has centered on outcomes such as early post-burn mortality, length of hospital stay, utilization of in-hospital resources (e.g., ICUs, ventilators) and the occurrence of specific physical complications such as contractures, infections or heterotopic ossification. However, these measures primarily reflect in-hospital clinical effectiveness rather than true long-term outcomes [2,3].

The global reduction in burn-related mortality and morbidity [4,5] has highlighted the importance of psychological consequences, including post-traumatic stress disorder (PTSD), depression and anxiety [6,7,8]. Burn care is also associated with substantial economic burden: the annual cost of treating a patient with burn exceeds that of other medical conditions, such as stroke or HIV/AIDS, with permanent disability accounting for 44% of the total cost [9].

A 2006 review of fifty publications summarized the functional consequences of burns using the International Classification of Functioning, Disabilities and Health (ICF) [10]. The ICF dimensions most frequently affected by burn injury were activities related to education and work (72%), mental function (70%), self-care (68%), relationships (62%) and mobility (60%) [10]. Nevertheless, some relevant functional outcomes received little to no attention, including heat sensitivity, skin elasticity, cardiovascular and digestive sequelae and visual, auditory and voice functions [2].

More recently, research examining psychosocial outcomes, such as health-related quality of life (HRQoL) after burns, has reflected a growing interest in a holistic approach to recovery. Despite some progress, the literature remains limited in this field [1,2].

The aim of this paper is to review the existing literature on the different aspects of quality of life in burn survivors post-discharge, with a particular focus on the temporal dynamics of recovery patterns. Given the multidimensional and heterogeneous nature of post-burn HRQoL research—encompassing physical, psychological, and social domains across diverse study designs and outcome measures—a narrative review methodology was selected to allow integrative thematic synthesis and interpretative discussion rather than quantitative aggregation.

## 2. Methodology

The narrative review methodology was selected because of the heterogeneity of post-burn HRQoL research, including differences in study design, populations, outcome measures and follow-up periods, which limited the suitability of quantitative synthesis. A targeted literature search of PubMed was conducted to identify clinically and conceptually relevant studies addressing HRQoL in adult burn survivors. The initial search was limited to papers published in English within the last 10 years and involving adults (19+ years). After this preliminary search, we excluded manuscripts not written in English, unsuitable study types (case reports, case–control studies, editorials) and manuscripts on other subjects unrelated to the study objective. Clinical trials, systematic reviews, narrative reviews and meta-analyses were studied and reference follow-up was conducted to identify other relevant papers not included in the initial search results. Older landmark studies and selected studies including pediatric populations were retained when they provided historically important data, foundational conceptual frameworks or long-term follow-up findings that remain frequently referenced in the burn rehabilitation literature. These studies were used selectively to contextualize temporal trends and evolution of HRQoL concepts. The literature was synthesized thematically into physical, psychological and social domains based on recurring patterns identified across the reviewed studies. Temporal recovery trajectories were subsequently examined across these domains to explore longitudinal patterns of adaptation and impairment.

The thematic framework of physical, psychological and social domains was initially informed by established HRQoL models and refined during the literature review as recurrent themes emerged across studies. Findings were synthesized descriptively and comparatively, with attention to variability between populations, healthcare settings and outcome measures.

Characteristics, outcome measures and principal findings of the included studies were systematically extracted and summarized in Table 1.

## 3. The Concept of Quality of Life Post-Burn

The World Health Organization (WHO) defines health as a state of complete physical, mental and social well-being, rather than merely the absence of disease or sickness [35]. This definition has been criticized for the use of the term “complete”, which refers to undefined and potentially infinite variables. Thus, quality of life (QoL) has been proposed as a more practical and operational concept, emphasizing the individual’s satisfaction with life and interconnection with their environment [2]. The WHO defines QoL as a person’s perception of their position in life within the context of the culture and value systems in which they live and in relation to their goals, expectations, standards and concerns [36]. This definition has been adopted in injury outcomes research to capture the patient’s perspective on recovery across physical, psychological and social domains [1]. Despite growing attention to QoL, no universally accepted measurement tool exists for burn survivors. Consequently, multiple HRQoL instruments have been adopted to better characterize patient recovery and guide individualized aftercare [16].

Historically, psychosocial adjustment after burns was assessed using psychometric measures and interviews [37]. However, the lack of standardization hindered statistical analysis, prompting the development of assessment tools [38,39] that meet the criteria of reliability, validity and responsiveness outlined by the Scientific Advisory Committee of the Medical Outcomes Trust [40]. Both generic and disease-specific instruments are available, each with distinct advantages. Disease-specific tools focus on issues particularly relevant to burn survivors, while generic instruments facilitate comparisons with other patient populations and normative data [2].

Most studies on post-burn QoL employ a combination of generic and burn-specific tools, with the Burn-Specific Health Scale-Brief (BSHS-B), Short Form-36 (SF-36) and EuroQoL 5-dimensions questionnaire (EQ-5D) being most commonly used (Table 2). Using both types of instruments is recommended to fully capture the impact of injury, although not all tools have been formally validated in burn populations [16].

A 2018 systematic review of HRQoL after burns revealed that only 34% of studies used longitudinal designs with multiple assessment points, which varied widely. The most common assessment times were during hospital admission and at 3, 6, 12 and 24 months post-injury. Although evidence suggests continued improvement in HRQoL after two years, high attrition rates complicate long-term follow-up [16].

Recent studies involving burn survivors evaluate both the overall QoL and key domains, including physical (pain and itching, fatigue and exercise, appearance), psychological (the presence of anxiety, depression, PTSD, personality changes, coping behaviors, post-traumatic growth, substance abuse, suicidal thoughts) and social aspects (perceived stigma, social support, community integration, family environment) [14].

### 3.1. Physical Dimension

Physical sequelae remain central determinants of quality of life after burn injury. Although acute wound closure represents a major milestone in recovery, many survivors continue to experience long-term symptoms affecting sensation, mobility, thermoregulation and appearance. These impairments often interact, amplifying disability and influencing psychosocial adjustment [34].

#### 3.1.1. Pain and Pruritus

Chronic pain and pruritus have a high prevalence even years after burn injury, substantially affecting work, relationships and quality of life. Chronic pain post-burn injury has been reported in approximately one-third of burn survivors [27,34,41,42]. In adult cohorts followed more than 10 years post-injury (with burns occurred during childhood or adulthood), over half reported chronic pain, nearly half reported pain interfering with daily life and pruritus was reported by 80% of patients [34]. The evolution pattern is different for various complaints: while pain and chronic persistence of wounds tend to decrease over time, skin fragility tends to increase over time [34].

Post-burn pain often presents with neuropathic characteristics, including burning, stabbing, electric and shooting sensations. In a retrospective review of 72 burn survivors, neuropathic-like symptoms were first reported on average 4.3 months post-injury [27]. Neuropathic mechanisms include nerve entrapment in scar tissue and neuroma formation, with histological evidence showing increased nociceptive nerve fibers within burn scars [27,41].

Post-burn pain is shaped by both physical and psychological factors. Movement, fatigue, temperature changes, stress, expectations and mood may intensify pain perception and contribute to functional limitation and reduced HRQoL [43].

Pain or discomfort consistently emerges as the most affected domain of functioning in burn survivors, regardless of burn severity. In patients with severe burns, 64% report problems related to pain or discomfort, compared with 29% of those with minor burns. Longitudinal data indicate that these complaints frequently persist, with 42% of patients reporting mild to severe pain at 12 months post-injury [30].

Persistent pain affects several areas of daily life, including work, social activities, sleep and concentration. It has also been associated with delayed or unsuccessful return to work, particularly when pain intensity remains moderate or severe [27]. Chronic pain after burn injury has been associated with larger burn size and the need for skin grafting, suggesting that injury severity plays a role in its development [27].

Pain perception is influenced not only by injury-related factors but also by cognitive, emotional and contextual factors such as attention, appraisal, expectations, past experiences and mood. Anticipatory anxiety and pain catastrophization may intensify perceived pain and hyperarousal states related to trauma may further amplify the experience [42].

Virtual reality (VR) has been used as an adjunct in burn care, primarily for pain management during procedures and rehabilitation. Based on the gate control theory of pain, VR may reduce procedural pain by redirecting attentional resources away from nociceptive stimuli [21].

#### 3.1.2. Motor Function

It has become standard practice to assess the impact of diseases and injuries on population health using composite outcome measures such as quality-adjusted life years (QALYs) and disability-adjusted life years (DALYs). These metrics combine years of life lost due to premature death with years lived with disability, weighted by severity, into a single summary measure [10].

Limitations in range of motion are reported to still be present in approximately 20% of patients with burns five years after the initial injury and even small burns have significant functional consequences when they concern areas such as the hands [10].

Long-term functional impairment after burn injury is frequently related to scar contractures, which are associated with reduced range of motion, may restrict joint mobility, cause pain or pruritus and interfere with daily activities, thereby contributing to disability and reduced HRQoL [11,19]. At discharge, approximately one-third of patients may present contractures, most often involving the shoulder, elbow, wrist, ankle and knee [11]—and those may persist at 12 months, particularly in operated joints (although reported prevalence varies across studies due to differences in definitions, timing of assessment and patient populations) [19].

Sets of predictors have been found for contracture development in general (male sex, black race, Hispanic ethnicity, medical problems, neuropathy, TBSA burned and TBSA grafted), for number of contractures (male sex, medical problems, flash burn, neuropathy, TBSA burned and TBSA grafted) and for contracture severity (male sex, black race, medical problems, neuropathy, TBSA burned and TBSA grafted) [11]. However, these associations may partly reflect disparities in access to rehabilitation, socio-economic factors and differences in healthcare utilization.

Although studies suggest that limited ROM at discharge has a low predictive value for long-term contracture, they indicate that joints with contractures at 12 months had often already shown restricted ROM at discharge that persisted over time [19,44]. The incidence of contracture in specific anatomical areas, such as fingers, may vary and can be influenced by treatment patterns and barriers to care [19].

Scarring also contributes to functional and psychosocial outcomes. Poor scar quality has been associated with reduced QoL, daily activity limitations, sleep disturbance, anxiety, depression and social difficulties [24]. Risk factors reported in the literature include younger age, larger and deeper burns, upper-limb or neck involvement, multiple surgical procedures and meshed grafts.

Hypertrophic scarring remains common and scar contracture can develop at multiple anatomical sites. No single method of scar control is universally effective. Progressive physical interventions such as dynamic splints, serial casting or static progressive splints may be more effective than multimodal approaches, including only massage, exercise and pressure. Patients with extensive scarring may require reconstructive surgery, with techniques such as Z-plasty for thin bands or release and grafting for thicker contractures. Individuals with darker skin tone have a greater tendency toward hypertrophic scarring. Long-term sequelae may include persistent itching decades after injury and altered sensation, particularly following deeper burns and skin grafting [34].

Despite aggressive early physical and occupational therapy, a significant proportion of patients develop functionally limiting contractures. Surgical correction using grafting, tissue rearrangement or local and distant flaps is often required but is associated with recurrence risk, cosmetic concerns, prolonged rehabilitation, increased financial burden and delayed return to work. Contributing factors to contracture formation include injury-related factors (depth, extent, cause, location), patient-related factors (genetic background, race, age, sex, nutritional status, compliance) and treatment-related factors (wound closure methods, timing, prevention strategies). Larger burns that cross multiple joints and require multiple operations, prolonged immobilization or extended ICU stays are associated with higher contracture incidence [11].

Functional consequences of scars and contractures are particularly evident in hand involvement. In patients with burned TBSA > 50%, hand function and total full-thickness injury have been identified as independent predictors of physical HRQoL. Hand burns requiring grafting are associated with a reduction in the physical component summary score. These findings support the need for early debridement, grafting and mobilization of the hand, although evidence-based prioritization of anatomical regions and graft types remains limited [2].

Rehabilitation strategies may improve functional outcomes. VR-based hand and wrist rehabilitation has demonstrated improvements in hand function, QoL and work performance. VR systems simulate daily activities and provide feedback, enabling individualized and task-specific training. Improvements in scar thickness and texture have also been observed, possibly due to increased active movement and scar stretching. However, grip and pinch strength may not significantly improve when strength training components are insufficient [21].

#### 3.1.3. Appearance

Self-esteem has been conceptualized as the discrepancy between the perceived self and the ideal self, with greater differences linked to negative attitudes toward life and difficulties in coping with adversity [18]. Appearance concerns are common among burn survivors, affecting up to 43% of patients, including those with minor burns [10].

Long-term studies have reported persistent sequelae that contribute to body image dissatisfaction. These include hypertrophic scarring (92% in grafted areas and 38% in non-grafted areas), decreased sensation in grafted regions (71%), hyperpigmentation in grafted areas (53%), fingernail deformities (35%) and skin breakdown (32%) [34]. Burn-related discomfort with body perception may arise from scar tissue, amputations or fear of functional loss. Negative societal reactions, depression, guilt and challenges in interpersonal relationships further exacerbate impaired body image [45].

Social support plays a protective role in mitigating stress and is directly associated with higher satisfaction with appearance, greater self-esteem and improved life satisfaction [26]. Importantly, social participation and body image influence each other over time, with early social support possibly contributing to later social participation indirectly through its effect on satisfaction with appearance [26].

### 3.2. Psychological Dimension

#### 3.2.1. Mental Health

A substantial proportion of burn survivors have a history of psychiatric disorders, which increases the risk of post-burn psychological problems. In some studies, two-thirds of survivors reported a lifetime psychiatric history [33], while in a cohort of 72 patients in the burn unit, 35% had documented pre-existing mental health conditions and 63% required ongoing psychological support for anxiety and depression following their injury [2]. At the same time, positive psychological traits such as optimism, extroversion, hope and belief in personal influence over outcomes were linked to improved long-term adjustment [2].

Depression is common in the aftermath of burn injuries, although its prevalence varies widely. Within the first month post-burn, rates as high as 53% have been reported, decreasing to between 13% and 35% at 12 months. Some of these cases may reflect pre-existing psychiatric vulnerability rather than new-onset depression [12]. Early recognition of depressive symptoms is therefore important, as they interact with other aspects of psychological recovery.

Post-traumatic growth (PTG) has been described as occurring alongside post-traumatic stress, rather than as its opposite. PTG can take two primary forms: a transformed understanding of the self and the world and the adoption of adaptive coping strategies, including humor, positive reframing, gratitude, downward comparison and meaning-making. It emerges following a disruption of core beliefs and may be facilitated by deliberate cognitive processing of the trauma [12]. Studies suggest a curvilinear relationship between depression and PTG, in which minimal depressive symptoms have little effect on growth, but increasing depressive severity is associated with reduced PTG. Stress has a linear, positive correlation with PTG, with higher perceived growth reported by individuals undergoing greater stress. As recovery progresses and the mental health of the patients improves, the need for PTG-related coping mechanisms decreases, which may contribute to lower Post-traumatic Growth Inventory scores. This trajectory of PTG may suggest the return to a new normal. One prospective study found that by three years post-burn, post-traumatic growth levels had stabilized even for patients with severe burns [12].

Beyond quantitative measures, qualitative research highlights additional positive outcomes in burn survivors, including enhanced life meaning, improved relationships, spiritual development and strengthened self-understanding. PTG is often conceptualized in three domains: changes in life philosophy, changes in self-perception and improved interpersonal relationships. Social support has been identified as a key environmental factor that facilitates PTG by promoting adaptive coping and personal evolution, with direct associations demonstrated between social support and PTG [28].

Cultural and demographic factors may further influence PTG. In a study of Iranian burn survivors, PTG levels were moderate to high and positively correlated with both total body surface area burned and educational level. Spiritual well-being was also significantly associated with higher PTG, suggesting that cultural background may shape how PTG is expressed, as Eastern populations often report higher growth than Western cohorts. However, the cross-sectional design and limited generalizability of these findings warrant cautious interpretation [28].

Burn survivors employ a variety of coping strategies to facilitate adaptation. Humor has been described as a central mechanism, enabling individuals to discuss their injuries without discomforting others and supporting social reintegration [13]. Empathy often develops following burn injury, with survivors reporting increased understanding, forgiveness, concern for others and a desire to assist future patients with burns. Functional adjustment varies between individuals and is not solely determined by the size or location of burns [13].

Return to productive activity is closely associated with perceived quality of life. Survivors who were physically able to resume work reported better outcomes than those unable to do so. A strong sense of self-efficacy supported reintegration into home and community roles, emphasizing its importance as a determinant of recovery [13].

Cognitive processing and re-examination of self-image also contribute to reconstructing the worldview necessary for PTG. Survivors described altered perspectives on the future, strengthened family commitments, enhanced spirituality, gratitude and sustained use of humor. Over time, PTG appeared to stabilize as the injury became integrated into a new sense of normality. Gratitude, in particular, emerged as a protective factor, supporting positive affect and overall well-being [13].

#### 3.2.2. Satisfaction with Life

Satisfaction with life (SWL) is a broad indicator of overall well-being that integrates physical, psychological and social dimensions. Among burn survivors, SWL recovery does not follow a uniform pattern. Research has identified two distinct trajectories: one group maintains relatively stable levels of life satisfaction over time, while another exhibits persistently low and declining SWL during the two years following injury. Approximately 60% of patients fall into the stable group, whereas around 40% experience deterioration [17].

The extent of total body surface area burned shows an inverse relationship with physical and psychological quality of life domains, while social and environmental domains appear less directly affected. This may reflect the more personal nature of physical and psychological adaptation compared with socially mediated domains [32]. Temporal patterns also suggest that psychological and physical domains are more affected in the early post-injury period, whereas social domain deterioration may emerge later as support systems change; however, causal conclusions are limited by the cross-sectional design of most studies [32].

Demographic and clinical characteristics may partially explain heterogeneity in SWL recovery. Factors such as mental health status, functional ability, ICU stay, amputation, marital status and appearance-related concerns have been associated with individual trajectories [17]. Members of the dissatisfied trajectory group reported lower initial SWL and continued decline over time, with risk factors including older age, male gender, dissatisfaction with appearance, prior psychiatric treatment or below-average mental health at discharge and unemployment prior to injury. All of these findings support the necessity of early identification of high-risk individuals [17].

Long-term health-related quality of life (HRQoL) is predicted by length of hospital stay (LOS), gender and age at injury. LOS appears to be a stronger predictor than TBSA, likely because it reflects not only injury severity but also associated medical and psychosocial complications. Older age and female gender have been associated with lower EQ-VAS (EuroQoL-Visual Analog Scale) scores [30]. Large multicenter studies support that SWL remains significantly lower than in non-burned populations for up to two years post-injury, with little improvement between 6, 12 and 24 months. Various demographic and clinical factors, including TBSA, LOS, school status and substance use, predict SWL at different time points [22].

Psychological factors such as self-efficacy and resilience also influence SWL. Self-efficacy suggests a stronger association with quality of life than resilience alone, although resilience contributes to the recovery process [13].

### 3.3. Social Dimension

#### 3.3.1. Return to Work

Rehabilitation aimed at facilitating return to work (RTW) is a central component of post-burn recovery. Across studies, the mean time to RTW varies, with one survey of 234 employed burn survivors reporting 14.3 weeks and another cohort showing 24.1 weeks, potentially influenced by differences in universal medical insurance coverage [9]. Multidisciplinary early rehabilitation programs increasingly recognize RTW as a critical outcome beyond traditional measures such as length of stay, Functional Independence Measurement (FIM) scores and discharge destination. Despite these efforts, systematic reviews indicate that 28–33.6% of burn survivors fail to return to work within 1–24 months post-injury, highlighting the persistent occupational burden associated with burns [29].

Return to work is influenced by multiple demographic, clinical and occupational factors. In the Netherlands, 54% of severe burns occur in individuals of working age, with one in five injuries happening at the workplace. Vocational evaluations frequently use the International Classification of Functioning (ICF) framework to assess body functions, activity limitations, participation restrictions and contextual factors affecting RTW. Predictors of RTW include age, comorbidities, psychiatric factors, pre-burn employment status, job characteristics, insurance coverage, TBSA (partial and full thickness), burn location (particularly hand and face), pain, surgical procedures, skin grafting and length of hospital stay [29].

The impact of burn injury on employment is substantial. At three months post-burn, 30% of previously employed patients remain absent from work, decreasing to 8% at one year, with similar rates at two years. Mean productivity loss is considerable, even in cohorts with relatively low mean TBSA (8%). Higher TBSA is consistently associated with increased absenteeism, while early absenteeism is also predicted by ICU admission, longer hospital stay and surgical treatment. Psychiatric comorbidities strongly correlate with prolonged or permanent work absence [29]. Psychological symptoms, including depression, anxiety and post-traumatic stress, affect nearly half of burn survivors and significantly influence social and employment outcomes. Approximately 28% never return to any form of employment and only 37% resume their pre-injury job without accommodations within 24 months [31]. These differences should be interpreted in light of methodological variability: Goei et al. [29] assessed return to work prospectively over two years, whereas Kazis et al. [31] focused on broader social participation outcomes, including work and employment. Thus, lower rates of return to the pre-injury job without accommodations are not directly equivalent to overall return-to-work rates.

Emerging interventions, such as virtual reality (VR)-based rehabilitation, have demonstrated effectiveness in improving quality of life, work performance, joint range of motion, functional scores, pain and anxiety without significant adverse effects. Given that joint dysfunction, particularly impaired hand function, has profound occupational consequences, VR-based hand and wrist rehabilitation may provide meaningful functional benefits comparable to those observed in stroke rehabilitation [21].

#### 3.3.2. Social Participation and Support

Social participation after burn injury remains challenging to assess due to the lack of comprehensive measurement tools. The Life Impact Burn Recovery Evaluation Profile (LIBRE) was developed to address this gap. It is a multidimensional, patient-reported instrument specifically designed to evaluate social participation in burn survivors, encompassing six domains: relationships with family and friends, social interactions, social activities, work and employment, romantic relationships and sexual relationships. The LIBRE Profile allows performance to be measured along a continuum from low to high functioning [31].

Social support exerts a protective influence on health and adjustment. High perceived support reduces vulnerability to illness and buffers stress by mitigating stressors, altering their meaning or promoting adaptive emotional responses. Among burn survivors, social support is a modifiable predictor of maladjustment and is positively associated with satisfaction with appearance, self-esteem, life satisfaction, social participation and physical activity [26].

## 4. Recovery Patterns of QoL in Adults After Burns

**Figure 1:**
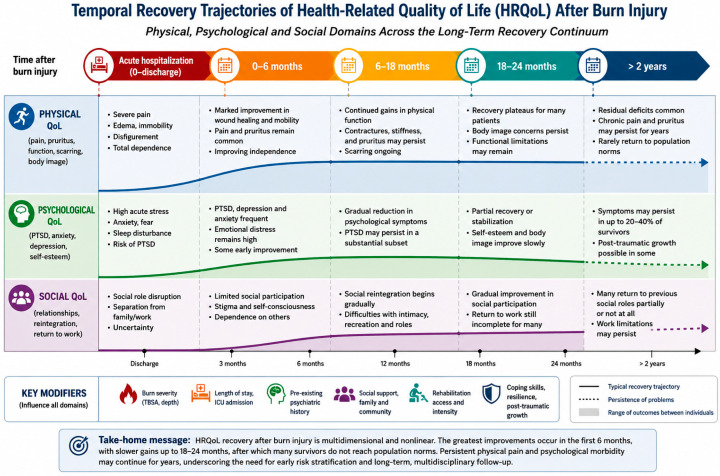
Overview of recovery patterns of quality of life in adults after burn injury. This synthetic schematic is based on longitudinal and trajectory evidence regarding physical, psychological and social recovery after burns, including Spronk et al. [20], Boersma-van Dam et al. [23], van Loey et al. [25], Ajoudani et al. [26], Goei et al. [29] and Kazis et al. [31].

Although several longitudinal studies and trajectory analyses have characterized HRQoL recovery during the first two years after burn injury in adults, no comprehensive multidimensional recovery model has been universally adopted [46,47]. Understanding how HRQoL evolves over time in adult patients with burns, considering factors such as age, gender, total body surface area burned, number of surgeries and length of hospital stay (LOS), provides essential guidance for clinical care [20,25]—as its synthesized in Figure 1.

Among the available longitudinal studies, the individual participant data meta-analysis by Spronk et al. provides one of the most comprehensive evaluations of HRQoL recovery trajectories in adult burn survivors, integrating data across multiple cohorts and follow-up intervals [20].

Analyses of the EQ-5D dimensions revealed that pain and discomfort are the most prevalent problems following burns. At baseline, nearly all patients with major burns and 89% of those with mild or intermediate burns reported pain and discomfort. Although improvements were observed over time, particularly during the first 12 months, pain and discomfort remained more common than issues in other domains and relative to the general population [20]. Other domains, such as mobility, self-care and usual activities, showed significant improvements over the first six months. For patients with mild and intermediate burns, difficulties with usual activities decreased from 81% at baseline to 30% at six months and 15% at 18 months, stabilizing thereafter at levels comparable to the general population. Patients with major burns improved to 40% at six months before plateauing. Self-care and mobility followed similar recovery patterns, with patients with major burns reaching normative levels later than those with less severe burns [20]. Anxiety and depression affected 30–40% of patients in the first month post-burn and remained elevated relative to the general population, particularly among major burn survivors [20].

EQ-5D utility scores tracked up to 24 months post-burn, adjusting for time since injury, age, gender, TBSA and LOS, demonstrated that the largest improvements occurred within six months, after which scores plateaued. Estimated values increased from 0.27 at baseline to 0.80 at 24 months, remaining below the population norm of 0.856. Recovery was slower among females, patients with longer hospital stays and those with larger burn areas, though absolute differences between groups diminished over time [20]. Overall, the greatest gains in HRQoL typically occur within the first six months, with modest further improvements until 18 months. Most EQ-5D dimensions improved over time, except for pain/discomfort and anxiety/depression, which remained impaired for up to seven years in both mild/intermediate and major burn groups [20]. This temporal pattern is broadly consistent with prospective longitudinal evidence showing progressive HRQoL improvement over 18 months after burn injury, with slower or incomplete recovery among patients with more severe burns [25], as well as with studies using recalled pre-burn HRQoL, which indicate gradual post-injury recovery but persistent impairment in pain, anxiety and depression domains [23].

Longitudinal studies have examined individual HRQoL trajectories relative to recalled pre-burn levels. Retrospective assessment is often necessary because pre-burn HRQoL is rarely prospectively collected. Most studies indicate that HRQoL domains are initially impaired but recover over time, with the exception of anxiety, depression and pain. SF-36-based research on recalled pre-burn HRQoL shows declines post-injury followed by gradual recovery. Burn severity, as measured by hospital stay and number of surgeries and psychological factors such as PTSD, consistently predict HRQoL outcomes. PTSD is prevalent among burn survivors, with approximately 9% formally diagnosed, 15% exhibiting sub-threshold symptoms and up to 43% reporting symptoms one year post-injury. Severely burned patients generally return to pre-burn HRQoL later or remain at lower levels than less severely burned patients. Group-level analyses may obscure whether individuals return to their own pre-burn HRQoL rather than population norms [23].

Hospital-acquired complications (HACs) exert a significant negative impact on physical health in patients with burns, while mental health is less affected. Urinary tract infections (UTIs), renal failure and venous thromboembolism (VTE) reduce physical composite scores; furthermore, patients experiencing VTE may face secondary effects from treatment and ongoing interventions. C. difficile infection was the only complication associated with reduced mental composite scores, though the reasons remain unclear. These findings align with prior research indicating reduced physical function for at least a year post-burn, whereas mental function remains relatively stable. Reducing HACs is therefore critical not only to decrease hospital costs and LOS but also to improve long-term physical outcomes [15].

Psychosocial adjustment is dynamic during the first year post-discharge, with differences being measured between three temporal references: at discharge (T1), 6 months post-discharge (T2) and 1 year post-discharge (T3). Satisfaction with appearance (SWAP) declined in the first six months post-burn before increasing over the subsequent six months, consistent with the notion that individuals initially experience internal conflict regarding appearance changes, which gradually resolves. Cultural factors may influence this trajectory, as seen in Iranian participants whose SWAP scores reflected a heightened emphasis on appearance compared to previous research [26].

Social participation follows a parallel course with body image, decreasing from T1 to T2, whereas social support increased from T1 to T2 and stabilized thereafter, highlighting the evolving nature of recovery [26]. Participants in a three-wave analysis of variables reported no limitations prior to injury, experienced mild restrictions six months post-burn and then gradually improved. Early social participation is associated with better body image six months later, while body image mediates the impact of social support on subsequent social participation. These findings suggest that intrinsic factors, such as satisfaction with appearance, play a more central role in social participation than external support, offering important implications for post-burn interventions [26].

Long-term HRQoL up to two years post-burn is relatively well documented, though not all survivors reach a stable recovery by this time. Some domains, such as physical functioning, continue to improve beyond 24 months, whereas other issues, particularly participation limitations related to mental health, may emerge later. The timing of assessments is critical: studies with late initial measurements, such as two months post-discharge or six months post-burn, may fail to capture expected recovery patterns. Repeated evaluations beginning soon after discharge, for example, at two weeks, are necessary to fully understand recovery trajectories [10,30].

Taken together, the reviewed literature suggests a phased recovery trajectory following burn injury. The early post-discharge period (0–6 months) is predominantly characterized by physical recovery, pain burden, emotional distress and dependence in daily activities. The intermediate phase (6–18 months) is marked by gradual functional improvement and increasing social reintegration, although body image dissatisfaction and psychological symptoms may remain prominent. Beyond 18–24 months, recovery trajectories become increasingly heterogeneous: some survivors approach population norms, whereas others continue to experience persistent pain, psychiatric morbidity, participation restrictions and reduced satisfaction with life. This conceptual trajectory underscores the non-linear and individualized nature of post-burn recovery and highlights the need for long-term multidisciplinary follow-up. The broader implications of these temporal recovery patterns are discussed below.

## 5. Discussion

At discharge, burn survivors are rarely healed; more often, they become chronically ill patients. An overview of risk factors for poor outcomes may be found in Table 3.

Burn injuries produce complex and long-lasting consequences that span physical, psychological and social domains. Recovery is dynamic, influenced not only by injury severity and clinical factors but also by rehabilitation, social support and individual psychological resilience. Understanding these multidimensional outcomes is essential for guiding care, tailoring interventions and optimizing long-term quality of life for adult burn survivors.

Compared with previous reviews focusing primarily on functional impairment or isolated HRQoL domains [2,10,14], the present review emphasizes the interaction between physical, psychological and social recovery trajectories over time. Earlier reviews largely described post-burn deficits, whereas more recent literature increasingly conceptualizes burn recovery as a dynamic and multidimensional adaptation process influenced by rehabilitation access, psychosocial support and individual resilience factors [12,13,16,17,20,26]. Despite improvements in survival and acute care, persistent impairment in pain/discomfort and anxiety/depression domains remains a consistent finding across studies and healthcare systems [20,23,25,30].

Interpretation of the literature is complicated by substantial heterogeneity between studies, including differences in burn severity, healthcare systems, rehabilitation access, outcome instruments and timing of assessment [14,16,20,25]. Variability in HRQoL measures, particularly between generic and burn-specific tools, may partially explain inconsistencies in reported outcomes and recovery trajectories [14,16,39].

As this is a narrative review, the authors did not perform a formal risk-of-bias assessment. For this reason, the findings were not interpreted as having equal weight. Greater emphasis was placed on systematic reviews, meta-analyses and longitudinal cohort studies, particularly when their conclusions were consistent across different populations, settings or assessment tools. Findings from cross-sectional studies, smaller observational cohorts, qualitative research and mixed adult–pediatric samples were interpreted more cautiously. These studies were useful for understanding patient experience and generating hypotheses, but they were not treated as sufficient evidence for causal conclusions. Therefore, associations involving burn severity, psychological morbidity, social support, return to work and HRQoL should be read as associations unless supported by longitudinal data.

### 5.1. Physical, Psychological and Social Outcomes

The available evidence is not equally strong across all domains. Longitudinal HRQoL trajectories, pain/discomfort and return-to-work outcomes are supported by larger cohort studies, systematic reviews or meta-analyses. By contrast, topics such as post-traumatic growth, spirituality, body image, coping strategies and some aspects of social participation rely more often on cross-sectional, qualitative or culturally specific studies. These findings remain clinically relevant, but they should be interpreted with greater caution.

Persistent physical sequelae, including pain, pruritus, contractures and scarring, remain major determinants of post-burn quality of life. Pain and discomfort are among the most common long-term complaints, affecting daily functioning, sleep, concentration, social engagement and return to work [27,30]. Chronic pain often exhibits neuropathic characteristics, influenced by injury severity, cognitive and emotional factors and environmental triggers [27,41,42]. Non-pharmacological interventions, such as virtual reality (VR)-based rehabilitation, show promise for pain management and functional recovery, although evidence remains limited [21].

Functional impairment persists, especially due to contractures and limited joint mobility, impacting both daily activities and occupational reintegration [11,19]. Early identification of risk factors—including TBSA, grafting and burn location—is critical for targeted interventions. Hand injuries disproportionately affect physical HRQoL, underscoring the importance of early mobilization, debridement and rehabilitation [2,21]. Scarring and altered appearance further influence physical and psychosocial well-being, with hypertrophic scars, pigment changes and sensory deficits contributing to body image dissatisfaction and reduced self-esteem [34,45].

Psychological sequelae—including depression, anxiety and post-traumatic stress disorder (PTSD)—remain prevalent and strongly influence overall QoL [20,31]. Depression is particularly common in the early post-burn period, although rates may reflect pre-existing vulnerability [12]. At the same time, post-traumatic growth (PTG) can emerge alongside distress, leading to adaptive coping strategies, improved self-understanding and strengthened relationships [12,13,28]. Self-efficacy and resilience are central to psychological recovery, influencing satisfaction with life and functional reintegration [13].

Social reintegration, especially return to work (RTW), is a key determinant of recovery. Despite rehabilitation efforts, 28–33% of survivors fail to resume employment within 24 months, highlighting the persistent occupational burden of burns [29]. RTW is influenced by demographic, clinical and occupational factors, including TBSA, burn location, psychiatric comorbidities, pain, ICU admission and social support [9,29]. Social support mitigates stress, enhances self-esteem, improves satisfaction with appearance and indirectly promotes social participation over time [26]. Patient-reported outcome measures capturing these domains are essential for individualized care planning [14,16].

### 5.2. Temporal Dynamics of QoL Recovery

HRQoL recovery follows a nonlinear trajectory and varies across domains. Physical and functional improvements predominantly occur within the first six months post-injury, with modest gains continuing up to 18 months before plateauing [20]. Pain and psychological symptoms may persist for years, particularly in individuals with severe burns, longer hospital stays or pre-existing psychiatric conditions [20]. EQ-5D utility scores indicate that, even at 24 months, HRQoL often remains below population norms, with slower recovery among females and those with larger burn areas. Hospital-acquired complications further impede physical recovery, reinforcing the importance of high-quality acute care [15].

Body image and social participation suggest interrelated trajectories. Early satisfaction with appearance correlates with subsequent social engagement, while social support reinforces this effect indirectly [26]. Temporal patterns show that satisfaction with appearance declines during the first six months after burn injury, corresponding to increased appearance-related dissatisfaction, and subsequently may improve as dissatisfaction diminishes during the following six months. These findings suggest that intrinsic factors, such as self-perception, play a central role in social reintegration, while external support acts as a facilitator.

### 5.3. Clinical and Research Implications

The evidence supports a holistic, multidimensional approach to burn care. Early identification of patients with a high risk of persistent pain, contractures, delayed RTW or psychological morbidity is essential for targeted interventions. Combining generic and burn-specific patient-reported outcome measures (PROMs) provides a comprehensive picture of patient experiences, informing personalized rehabilitation strategies [14,16]. VR-based rehabilitation and intensive hand therapy are promising interventions that can enhance functional and psychosocial outcomes [21].

Longitudinal follow-up beyond two years is needed to capture late-emerging mental health and social participation issues. Additionally, cultural and demographic factors are associated with post-traumatic growth and appearance-related outcomes, underscoring the importance of individualized assessment and culturally sensitive care [28]. Optimizing the timing and frequency of HRQoL assessments—starting soon after discharge and continuing at multiple intervals—is critical to accurately map recovery trajectories and guide interventions [10,30].

### 5.4. Limitations

We acknowledge several methodological limitations inherent to the narrative nature of this review. Specifically, our literature search was restricted to a single database (PubMed), and the study selection process lacks the methodological rigor of a systematic review and is therefore susceptible to selection and interpretative bias. Moreover, no formal risk-of-bias assessment was performed. The included literature was heterogeneous regarding study design, outcome measures, burn severity and follow-up duration, limiting direct comparison between studies, and as some conclusions were derived from observational and cross-sectional evidence, causal inference cannot be established. Finally, although the review focused primarily on adult populations, several historically important studies included mixed adult and pediatric cohorts, potentially limiting generalizability.

## 6. Conclusions

Throughout the body of evidence reviewed, a consistent message emerges: burns exert a profound and enduring influence on patients’ health-related quality of life. However, recovery trajectories vary across healthcare settings and cultural contexts, from differences in the influence of factors such as age and gender to differences in outcome scores and tools. This variability highlights the need for multidisciplinary care that extends beyond the acute phase, with systematic, longitudinal assessment using validated tools to better understand and support patients and their families.

## Figures and Tables

**Table 1:** Summary of included studies.

Author (Year)	Study Design	Sample Size	Population Characteristics	Outcome Measures	Key Findings
Goverman et al. (2017) [11]	National database study	1865	Adult burn survivors	Contracture assessment measures	Contractures remained highly prevalent after severe burns and were associated with total body surface area affected (TBSA) and grafting.
Martin et al. (2017) [12]	Prospective longitudinal study	73	Adult burn survivors	QoL and Posttraumatic Growth Inventory	PTG and QoL evolved dynamically during recovery and stabilized over time.
Abrams et al. (2016) [13]	Qualitative study	8	Adult burn survivors	Qualitative interviews	Survivors described humor, empathy and self-efficacy as key coping strategies.
Griffiths et al. (2017) [14]	Systematic review	Not available	Adult burn survivors	Patient-reported outcome measures	Identified BSHS-B, SF-36 and EQ-5D as the most commonly used instruments in burn research.
Deeter et al. (2019) [15]	Longitudinal cohort study	496	Adult burn survivors with hospital-acquired complications	Physical and mental composite QoL scores	Hospital-acquired complications negatively affected long-term physical QoL.
Spronk et al. (2018) [16]	Systematic review	Not available	Adult burn survivors	HRQoL instruments including SF-36, EQ-5D, BSHS-B	Recovery in HRQoL continued beyond 2 years, though longitudinal data were limited.
Amtmann et al. (2020) [17]	Longitudinal cohort study	3587	Adult burn survivors	Satisfaction with Life Scale	Approximately 40% of survivors demonstrated persistently low satisfaction with life trajectories.
Mehrabi et al. (2022) [18]	Systematic review	762	Patients with burn	Self-esteem and psychosocial measures	Appearance-related concerns and reduced self-esteem were common after burns.
Hendriks et al. (2022) [19]	Prospective cohort study	36	Adult and pediatric burn survivors in low-income countries	Joint function, disability and QoL assessments	Burn scar contractures significantly impaired joint function and quality of life.
Spronk et al. (2020) [20]	Meta-analysis	1687	Adult burn survivors	EQ-5D	Most HRQoL recovery occurred within 6 months, with pain and depression/anxiety persisting for up to 24 months.
Lan et al. (2023) [21]	Systematic review and meta-analysis	535	Adult and pediatric burn rehabilitation patients	Functional outcomes, QoL, pain and anxiety measures	Virtual reality rehabilitation improved QoL, ROM, pain and anxiety outcomes.
Goverman et al. (2016) [22]	Longitudinal cohort study	1129	Adult and pediatric burn survivors	Satisfaction with Life Scale	Satisfaction with life remained below population norms up to 2 years post-burn.
Boersma-van Dam et al. (2021) [23]	Longitudinal cohort study	309	Adult burn survivors	SF-36 and retrospective pre-burn HRQoL measures	Psychological factors and PTSD significantly influenced long-term HRQoL recovery.
Moi et al. (2006) [6]	Observational study	95	Adult burn survivors	Generic HRQoL measures	Survivors reported impaired generic health status despite perceiving good overall quality of life.
Finlay et al. (2017) [24]	Observational study	341	Adult and adolescent burn survivors	Modified Vancouver Scar Scale (mVSS), QoL measures	Poor scar quality correlated significantly with reduced quality of life.
van Loey et al. (2012) [25]	Prospective cohort study (multicenter)	260	Adult burn survivors	HRQoL assessments over 18 months	HRQoL improved over time but severe burns recovered more slowly.
Ajoudani et al. (2018) [26]	Longitudinal cross-lagged study	100	Adult and adolescent burn survivors during early rehabilitation	Social participation, social support and body image measures	Social support positively influenced body image and later social participation.
Nedelec et al. (2016) [27]	Case series	17	Adult burn survivors with neuropathic pain	Somatosensory rehabilitation outcomes	Neuropathic pain symptoms commonly emerged several months post-burn and affected QoL.
Ajoudani et al. (2019) [28]	Cross-sectional study	102	Adult Iranian burn survivors	PTG and spirituality measures	Social support and spirituality were positively associated with post-traumatic growth.
Goei et al. (2016) [29]	Prospective cohort study	104	Adult burn survivors	Return-to-work and cost assessments	A substantial proportion of survivors failed to return to work within 24 months.
Spronk et al. (2019) [30]	Multicenter cross-sectional study	256	Adult burn survivors with minor and severe burns	EQ-5D	Pain/discomfort remained one of the most impaired domains long-term.
Kazis et al. (2017) [31]	Prospective cohort study	601	Adult burn survivors	Social participation measures: relationships with family and friends, social interactions, social activities, work and employment, romantic relationships and sexual relationships.	The development of the Life Impact Burn Recovery Evaluation Profile (LIBRE), a patient-reported multidimensional assessment tool for understanding social participation after burn injuries.
Gautam et al. (2022) [32]	Cross-sectional study	150	Adult burn survivors	WHOQOL domains	Larger TBSA was associated with poorer physical and psychological QoL domains.
Dyster-Aas et al. (2008) [33]	Observational study	73	Adult burn survivors	PTSD and depression measures	Pre-existing psychiatric morbidity increased risk of post-burn depression and PTSD.
Holavanahalli et al. (2010) [34]	Long-term follow-up study	98	Adult burn survivors (with burns occurred during childhood or adulthood)	Skin-related outcomes and QoL measures	Chronic pain, pruritus, hypertrophic scarring and sensory disturbances persisted years after injury.

**Table 2:** Common HRQoL Assessment Tools Used in Burn Research.

Instrument	Type	Domains Assessed	Strengths	Limitations	Burn-Specific Validation
**SF-36**	Generic	Physical and mental health	Widely validated, population comparison possible	Less sensitive to burn-specific issues	Partial
**EQ-5D**	Generic	Mobility, self-care, pain/discomfort, anxiety/depression	Utility scoring, longitudinal tracking	Ceiling effects in mild burns	Partial
**BSHS-B**	Burn-specific	Heat sensitivity, body image, work, affect	Sensitive to burn-related concerns	Less useful for general population comparison	Yes
**LIBRE Profile**	Burn-specific	Social participation	Strong social reintegration focus	Less data on long-term trajectories	Yes
**SWAP**	Appearance-specific	Body image dissatisfaction	Sensitive to appearance-related distress	Narrow scope	Partial

**Table 3:** Risk factors for poor outcomes.

Category	Risk Factor	Outcome Affected
Clinical	ICU admission	Delayed return to work, poorer physical recovery
Clinical	Facial burns	Body image dissatisfaction, social reintegration difficulties
Clinical	Hospital-acquired complications (e.g., UTI, VTE, renal failure)	Reduced physical HRQoL
Clinical	Full-thickness burns	Functional limitation, scarring, poorer physical outcomes
Clinical	Burn depth and graft requirement	Chronic pain and scar severity
Clinical	Larger TBSA burned	Reduced physical HRQoL, delayed recovery, increased pain and contracture risk
Clinical	Longer length of hospital stay (LOS)	Lower long-term HRQoL, delayed recovery, poorer EQ-5D scores
Clinical	Multiple surgical procedures/grafting	Chronic pain, scarring, contractures, reduced physical QoL
Clinical	Hand burns	Reduced hand function, impaired occupational reintegration, poorer physical QoL
Clinical	Contractures	Reduced ROM, disability, impaired daily functioning
Clinical	Hypertrophic scarring	Poor body image, anxiety, reduced QoL
Clinical	Neuropathic pain	Sleep disturbance, impaired work and social functioning
Demographic	Black race/Hispanic ethnicity	Higher contracture risk in some studies
Demographic	Female sex	Slower HRQoL recovery and lower EQ-VAS scores
Demographic	Male sex	Increased contracture burden in some cohorts
Demographic	Older age	Lower HRQoL and slower recovery
Psychological	Pain catastrophization	Increased perceived pain and hyperarousal
Psychological	Low resilience/coping ability	Poor psychological adaptation
Psychological	Low self-efficacy	Poor adjustment and reduced return to work
Psychological	Pre-existing psychiatric illness	Depression, PTSD, impaired adjustment
Psychological	Depression	Reduced satisfaction with life, impaired social reintegration
Psychological	Anxiety/PTSD symptoms	Persistent psychological impairment, reduced QoL
Psychological	Poor self-esteem/body image dissatisfaction	Reduced social participation and QoL
Social	Unemployment prior to injury	Lower satisfaction with life trajectory
Social	Occupational physical demands	Delayed RTW and functional limitations
Social	Social stigma/self-consciousness	Isolation and reduced participation
Social	Limited social support	Lower self-esteem, poorer body image, reduced social participation
Social	Failure to return to work	Reduced QoL and social reintegration

## Data Availability

No new data were created or analyzed in this study. Data sharing is not applicable to this article.

## Abbreviations

The following abbreviations are used in this manuscript:

BSHS-B	Burn-Specific Health Scale-Brief
DALYs	Disability-Adjusted Life Years
EQ-5D	EuroQoL 5-Dimensions Questionnaire
EQ-VAS	EuroQoL-Visual Analog Scale
FIM	Functional Independence Measurement
HACs	Hospital-Acquired Complications
HRQoL	health-Related Quality of Life
ICF	International Classification of Functioning, Disabilities and Health
LIBRE	Life Impact Burn Recovery Evaluation Profile
LOS	Length of Hospital Stay
mVSS	Modified Vancouver Scar Scale
NHS	United Kingdom National Health Service
PROMs	Patient-Reported Outcome Measures
PTG	Post-Traumatic Growth
PTSD	Post-Traumatic Stress Disorder
QALYs	Quality-Adjusted Life Years
QoL	Quality of Life
ROM	Range of Motion
RTW	Return to Work
SF-36	Short Form-36 Questionnaire
SWAP	Satisfaction with Appearance
SWL	Satisfaction with Life
TBSA	Total Body Surface Area
VR	Virtual Reality
VTE	Venous Thromboembolism
WHO	World Health Organization
